# Responsiveness to conspecific distress calls is influenced by day-roost proximity in bats (*Saccopteryx bilineata*)

**DOI:** 10.1098/rsos.160151

**Published:** 2016-05-18

**Authors:** Maria Eckenweber, Mirjam Knörnschild

**Affiliations:** 1 Evolutionary Ecology and Conservation Genomics, University of Ulm, Ulm, Germany; 2 Animal Behaviour Lab, Institute for Biology, Free University Berlin, Berlin, Germany; 3 Smithsonian Tropical Research Institute, Balboa, Panama

**Keywords:** distress calls, social relevance, location-dependent responsiveness, social call, Chiroptera

## Abstract

Distress calls signal extreme physical distress, e.g. being caught by a predator. In many bat species, distress calls attract conspecifics. Because bats often occupy perennial day-roosts, they might adapt their responsiveness according to the social relevance in which distress calls are broadcast. Specifically, we hypothesized that conspecific distress calls broadcast within or in proximity to the day-roost would elicit a stronger responsiveness than distress calls broadcast at a foraging site. We analysed the distress calls and conducted playback experiments with the greater sac-winged bat, *Saccopteryx bilineata*, which occupies perennial day-roosts with a stable social group composition. *S. bilineata* reacted significantly differently depending on the playback's location. Bats were attracted to distress call playbacks within the day-roost and in proximity to it, but showed no obvious response to distress call playbacks at a foraging site. Hence, the bats adapted their responsiveness towards distress calls depending on the social relevance in which distress calls were broadcast. Distress calls within or in proximity to the day-roost are probably perceived as a greater threat and thus have a higher behavioural relevance than distress calls at foraging sites, either because bats want to assess the predation risk or because they engage in mobbing behaviour.

## 1. Introduction

Distress calls appear in the vocal repertoire of different taxa and are defined as vocalization given in situations of extreme distress, like being caught by a predator or in a trap [1]. Distress calls have a similar acoustic design across taxa ([2]: birds, primates; [3]: rodents; [4]: deer; [5]: bats; [6]: frogs). They can be generally described as low-frequency calls, often with noisy parts and a large bandwidth, making them audible over a larger distance [2,7,8]. Distress calls are only produced under the physical stress of an actual attack (and are sometimes also called fear screams), whereas alarm calls or alert calls are used to warn conspecifics prior to an attack [9].

In birds, the usage of distress calls is well described, and studies offer a wide range of hypotheses on their function (see [1,10,11]). Distress calls can be produced to startle a predator into releasing its prey or to attract additional predators that might distract the first one [1,10,12,13]. Alternatively, distress calls can be directed at conspecifics to request aid and/or to initiate mobbing behaviour [14–16]. However, distress calls can also repel or warn conspecifics [17,18] or provide other individuals with information about the respective predator [10]. Obviously, none of the above-mentioned potential functions of distress calls is mutually exclusive. On the species level, the frequency in which distress calls are produced is related to both the predation risk and the probability of escape [11].

Several bat species are attracted to distress calls of conspecifics [19–23] or even heterospecifics from the same family [5,24]. However, as in birds, it is a matter of active debate whether the observed phonotaxis behaviour should be considered as evidence for mobbing or personal inspection/risk assessment [23]. Moreover, none of the studies so far considered whether the social context or relevance in which distress calls were presented influenced the bats' responsiveness. It is conceivable that predation events close to perennial day-roosts constitute a greater threat for bats, and thus have a higher relevance, than predation events in fluctuating foraging grounds. If this was the case, distress call playbacks in the vicinity of day-roosts might cause different (i.e. stronger and/or faster) behavioural responses than playbacks at foraging sites (where the majority of distress call studies in bats have been conducted). Alternatively, group hunting bat species [25,26] or species feeding on clumped resources such as fruiting trees might perceive predation events at foraging sites to be equally or even more relevant than predation events at the day-roost. This would explain the readiness with which frugivorous bats react to conspecific distress calls in a foraging context ([5,20], own observations). Taken together, the responsiveness of many bat species is probably influenced by the social context in which distress calls are produced. Responsiveness might increase or decrease depending on the perceived relevance of a predation event at the roost site or the foraging ground.

We chose the greater sac-winged bat, *Saccopteryx bilineata*, to study whether the social context of a given situation influences the responsiveness towards conspecific distress calls. This common neotropical bat lives in year-round stable colonies which can consist of up to 60 individuals. Social groups within colonies have a harem-like structure in which one harem male guards a territory of up to 2 m^2^ of vertical roosting area in which two to eight females roost [27]. Additionally, satellite males queuing for harem access are also often present [28]. *S. bilineata* roosts in well-lit cave entrances, large tree-cavities and on the outside walls of buildings [29]. Day-roosts can be occupied continuously for decades [30]. As an aerial insectivorous bat, *S. bilineata* hunts close to vegetation at forest edges and in forest gaps [31]. Adult individuals from the same colony apparently forage separately and do not use communal night-roosts [32]. *S. bilineata* has a large vocal repertoire with individual-, group- and sex-specific signatures in certain vocalization types [33–37]. Many adults and especially subadults readily produce distress calls when being handled after capture (personal observations 2012).

Given the natural history and foraging behaviour of *S. bilineata*, we hypothesized (i) that the bats would respond to conspecifics' distress calls with approach behaviour and (ii) that their responsiveness would depend on the perceived social relevance of a given situation. Specifically, we predicted that distress calls broadcast within the day-roost or in close proximity to it would trigger a greater responsiveness than distress calls broadcast at a foraging site.

## 2. Methods

### 2.1. Study site, period and animals

Playback experiments were performed in August 2012, August–September 2013 and July–August 2014 on Barro Colorado Island (BCI). This field station of the Smithsonian Tropical Research Institute is located on a 15.6 km^2^ island in the artificial Gatun Lake, Panamá (9°9′17 N, 79°51′53 W; 25–165 m above sea level). The natural environment of BCI, which is part of Barro Colorado Natural Monument (BCNM, 54 km^2^ in total), is a semi-evergreen, moist tropical lowland forest [38]. For this study, we worked with 11 different *S. bilineata* colonies (over 100 bats in total) which were all located on the outside walls of buildings belonging to the field station. Owing to a long-term project at the study site, *S. bilineata* were monitored and caught regularly and well habituated to the presence of observers.

### 2.2. Call recording and analysis

**Figure 1.**
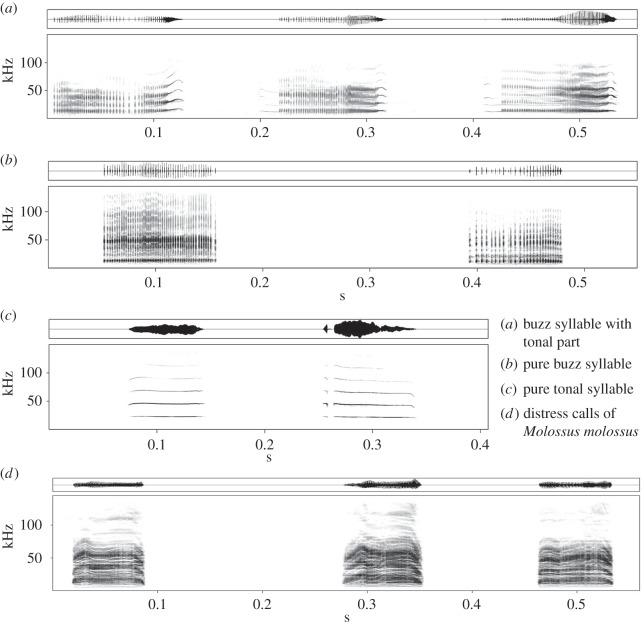
Three different distress call categories produced by handheld *S. bilineata* (*a*–*c*). Buzz syllables with tonal part (*a*) comprised the most common category, which we therefore used as stimuli for our playback experiment. As control, we used the distress calls of *M. molossus* (*d*). Spectrograms were created with a 1024-point FFT and a Hamming Window with 87.5% overlap (frequency resolution: 488 Hz, time resolution: 0.256 ms).

The distress call recordings for our study were made during routine catching events in 2011, 2012 and 2014. We used a high-quality ultrasonic recording set-up (500 kHz sampling rate and 16-bit depth resolution) to record the distress calls from handheld bats. The set-up consisted of an ultrasonic microphone (Avisoft USG 116Hm with condenser microphone CM16; frequency range 1–200 kHz ± 3 dB) connected to a laptop computer (Viliv, UltraMobil PC) running the software Avisoft-Recorder v. 4.2 (R. Specht, Avisoft Bioacoustics, Glienicke, Germany). To obtain a basic acoustic description of distress calls in *S. bilineata*, we analysed a subset of our recordings (1239 distress calls from 54 individuals recorded in 2014) with the software Avisoft SASLab Pro v. 5.2.07 (R. Specht, Avisoft Bioacoustics). Only distress calls with excellent signal-to-noise ratio were selected for acoustic analysis. We discriminated visually between three different distress call categories: buzz syllables with a tonal part, pure buzz syllables and pure tonal syllables (figure 1). We measured 26 buzz syllables with a tonal part from 13 bats, 25 pure buzz syllables from 11 bats and 25 pure tonal syllables from 13 bats. In each syllable (or, in the case of buzz syllables with tonal parts, in each syllable part), we measured one temporal parameters (duration) and three spectrum-based parameters (peak frequency, bandwidth and entropy). The three spectrum-based parameters were averaged over the entire syllable as well as measured at the start, centre and end of each syllable. Thus, we measured 13 acoustic parameters per syllable (or syllable part). We measured all spectrum-based parameters in the harmonic with the highest recording quality and subsequently converted all measurements to the first harmonic which normally had the highest sound pressure level. Start and end of the syllables were defined visually based on the information in the oscillogram and the spectrogram. We created spectrograms with a 1024-point FFT and a Hamming Window with 87.5% overlap (frequency resolution: 488 Hz, time resolution: 0.256 ms).

### 2.3. Playback stimuli

We selected high-quality distress calls (category: buzz syllables with tonal part) from eight *S. bilineata* individuals. As a control, we used distress calls from eight *Molossus molossus* individuals, a sympatric bat species inhabiting the same buildings as *S. bilineata*. We did not use noise as a control, because a previous study found that noise stimuli repel *S. bilineata* ([23], M.K., own observation). All playback stimuli were bandpass filtered and normalized (to 100%). Each playback file was 30 s long and consisted of 25 different distress calls from a single stimulus donor (either *S. bilineata* or *M. molossus*) interspaced by silence of 0.1–3.3 s. All donors were used several times in playback sessions (19 sessions in total): five of the donors were used in two playback sessions, and three donors were used in three playback sessions. Stimulus donors never belonged to the tested colony. For each playback session, the same conspecific and heterospecific stimulus donors were used during all three consecutive playback trials (see below). Playback files were generated with Cool Edit 2000 (Syntrillium, Phoenix, AZ).

### 2.4. Playback design

We tested the reaction of *S. bilineata* to conspecific distress calls and heterospecific distress calls (as control) in three consecutive playback trials that were broadcast within the roost, in close proximity to the roost and at a foraging site. All three playback trials were conducted in a paired design during the same playback session (either at dusk or dawn) in order to reach the same group of bats during all playback trials. Each playback trial consisted of a separate conspecific and heterospecific playback file that were broadcast in direct succession to one another (in a balanced order). Each conspecific and heterospecific playback had a total duration of 90 s and was divided into three 30 s periods: pre-observation (silence), playback period (with 25 distress calls of a single *S. bilineata* or *M. molossus* donor) and post-observation period (silence). Playback sessions were only considered to be valid when all three playback trials were completed successfully, namely when *S. bilineata* were present (i.e. detectable by echolocation calls) throughout all trials. Playback stimuli (300 kHz sampling rate and 16-bit depth resolution) were broadcast with an ultrasonic speaker (Avisoft UltraSoundGate Player BL Pro, single speaker version; 5–70 kHz ± 6 dB) connected to a laptop computer (MSI, U100 series) running the software Avisoft-Recorder v. 4.2. (R. Specht, Avisoft Bioacoustics). The amplitudes of playback stimuli were adjusted to 55–60 dB SPL at a distance of 1 m.

As mentioned above, three playback trials (each consisting of conspecific and heterospecific distress call playbacks) were performed consecutively for each playback session at three different locations: (1) within the focal colony; (2) in close proximity, i.e. 3–5 m, to the focal colony; and (3) at a nearby foraging area, i.e. at 10–25 m distance from the day-roost. The speaker was placed on the ground in all three locations in order to mimic an attacked and/or caught bat. The order of playback trials per session was 3–2–1 at dawn and 1–2–3 at dusk, thus incorporating the natural activity pattern of our colonies. At dawn and dusk, immediately before entering or leaving the roost, *S. bilineata* often forage in the vicinity of their day-roost, flying back and forth in regular beats ([39], own observations).

During playback trials, we monitored the presence of *S. bilineata* (using echolocation calls as proxy) with the same equipment used for the sound recordings of playback stimuli. Over the course of three field seasons (2012–2014), we conducted 19 valid playback sessions (broadcasting both *S. bilineata* and *M. molossus* distress calls at three different locations) with 11 different colonies of *S. bilineata*. Eight of 11 colonies were tested in 2 years. Because colony compositions (i.e. the individuals present in a particular day-roost) differed between years, we considered playback trials in the same day-roost but in different years as separate samples for our analysis.

### 2.5. Playback analysis

The reactions to our playbacks were analysed acoustically with Avisoft SASLab Pro (v. 5.2.07, R. Specht). As reaction, we defined an increase of echolocation calls in the playback and post-observation period compared with the pre-observation period. We decided to count echolocation calls (identified after [31]) instead of whole passes, because *S. bilineata* is highly manoeuvrable (e.g. capable of hovering), and we did not want to underestimate their reaction by counting passes instead of calls (the latter increase in number when a bat is continuously in the vicinity of the speaker, whereas the former do not). All echolocation calls that exceeded a 50% threshold in the amplitude spectrum (oscillogram created with Avisoft SASLab Pro) were counted separately for each playback period (pre-observation, playback, post-observation). Thus, faint echolocation calls were not counted as a response to our playbacks (nevertheless, they were suitable to ensure that bats were present for our playback trials). Subsequently, we combined playback and post-observation period (as reaction period) by calculating the mean number of echolocation calls in the reaction period. The numbers of echolocation calls in pre-observation and reaction period were used as a proxy for ‘activity’. The difference between reaction and pre-observation period was a proxy for ‘responsiveness’ (high numbers indicate increased responsiveness and vice versa).

### 2.6. Statistics

We performed a paired *t*-test comparing the activity of *S. bilineata* during the pre-observation and the reaction period, to test whether bats reacted to our playbacks. Moreover, we ran a repeated-measures ANOVA to test whether the responsiveness to distress calls was dependent on where the playback was broadcast (within the roost, in proximity to it or at a foraging site). We used K–S tests to ascertain that the activity data and the standardized residuals of the ANOVA did not deviate from a normal distribution. All statistical tests were conducted in SPSS v. 20.0 (IBM Corporation, New York).

## 3. Results

### 3.1. Distress call occurrence and acoustic structure

The majority of captured bats emitted distress calls at some point during routine processing (64%; 87 out of 136 individuals, 69 females and 67 males). We found three different categories of distress calls in *S. bilineata* (figure 1 and table 1). The most common category consists of a noisy and a tonal part, resembling an exaggerated buzz syllable of the territorial song of *S. bilineata* males [33]. 55% of the 1239 distress calls we analysed belonged to this category. The second category (31% of all distress calls) was a pure buzz without a tonal part. The third and less used category (14% of all distress calls) was purely tonal (figure 1). The first two categories of distress calls were audible for humans, the purely tonal one was not.

**Table 1.** Acoustic parameters of the three distress call categories which *S. bilineata* produced when being handled. All measurements of spectrum-based parameters were done in the harmonic with the highest recording quality and subsequently converted to the first harmonic. Mean values with standard derivations are shown.

distress call category	*N*	duration (ms )	peak frequency (start) (kHz)	peak frequency (centre) (kHz)	peak frequency (end) (kHz)	peak frequency (mean) (kHz)	bandwidth (mean) (kHz)	entropy (mean)
buzz-tonal (buzz part)	26	120 ± 34	13.1 ± 2.6	12.6 ± 2.1	14.6 ± 2.0	13.2 ± 1.9	11.1 ± 2.7	0.18 ± 0.02
buzz-tonal (tonal part)	26	20 ± 9.6	14.8 ± 2.2	15.5 ± 3.0	14.4 ± 3.0	15.5 ± 3.0	3.8 ± 0.9	0.11 ± 0.01
pure buzz	25	166 ± 23.7	12.7 ± 1.9	13.2 ± 1.4	12.8 ± 1.0	13.4 ± 1.0	10.8 ± 1.7	0.18 ± 0.02
pure tonal	25	55.4 ± 34.8	23.1 ± 2.2	21.5 ± 1.2	19.3 ± 2.7	21.9 ± 1.3	7.1 ± 4.1	0.14 ± 0.04

### 3.2. Responses to playback experiment

**Figure 2.**
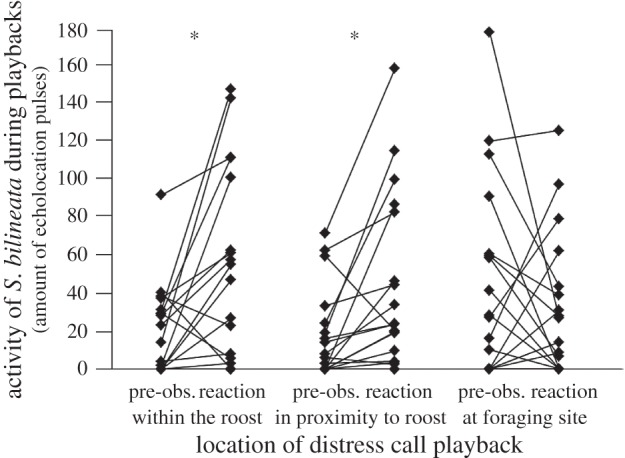
Activity (measured as total amount of echolocation calls per 30 s) of *S. bilineata* when conspecific distress calls were broadcast within the roost, in proximity to the roost, and at foraging site. Asterisk corresponds to *p* ≤ 0.01.

**Figure 3.**
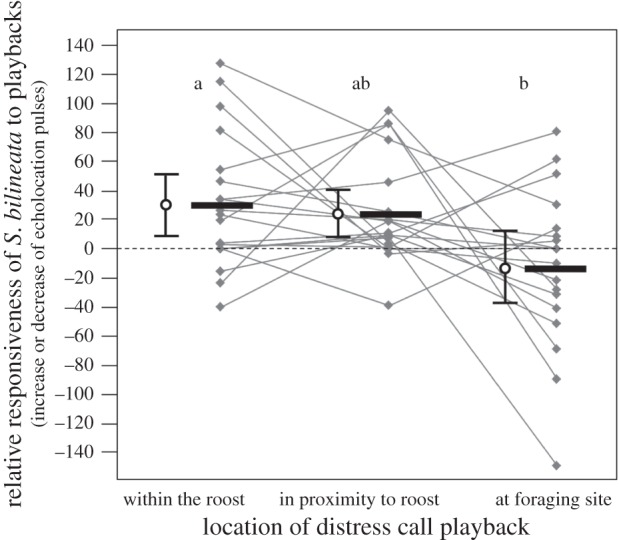
Relative responsiveness of *S. bilineata* to conspecific distress calls broadcast within the roost, in proximity to the roost and at a foraging site. Means (solid lines) with 2 standard errors (whisker plots) are depicted in black, the data points per colony (*n* = 19) in grey. Different letters depict a significant difference.

*Saccopteryx bilineata* significantly increased their activity when conspecific distress calls were broadcast within the roost or in proximity to it, but not at a foraging site (paired *t*-test comparing reaction period to pre-observation period; within: *t*_18_ = −2.901, *p* = 0.010; proximity: *t*_18_ = −2.990, *p* = 0.008; foraging: *t*_18_ = 1.014, *p* = 0.324; figure 2). In contrast to this, *S. bilineata* did not increase their activity in reaction to distress calls from the sympatric bat *M. molossus* (paired *t*-test; within: *t*_18_ = −0.387, *p* = 0.703; proximity: *t*_18_ = −0.139, *p* = 0.891; foraging: *t*_18_ = 0.142, *p* = 0.888), thus suggesting that *S. bilineata'*s reaction to distress calls is confined to conspecifics (see electronic supplementary material for the complete dataset). The responsiveness to conspecific distress calls was comparatively large when distress calls were broadcast in the roost or in close proximity to it (figure 3), but significantly reduced when distress calls were broadcast in the nearby foraging ground (repeated-measures ANOVA: *F*_2,17_ = 3.756, *p* = 0.045, *η*^2^ = 0.306; *post hoc*: within versus proximity: *p* = 0.999; within versus foraging: *p* = 0.039; proximity versus foraging: *p* = 0.100).

## 4. Discussion

The majority of *S. bilineata* distress calls are low frequency, and have noisy components and a large bandwidth, making distress calls audible for conspecifics and various hearing predators such as primates, coatis, raptors and toucans. Even though distress calls reliably elicited phonotaxis in nearby conspecifics, it is currently unclear whether conspecifics are the intended receivers (to warn them or call for their help) or whether distress calls are directed at the predator instead (to startle it or attract other predators to fend it off). Conspecifics could also eavesdrop on distress calls to selfishly inspect the potential source of danger. All scenarios have been suggested for avian distress calls [1,10,12–18] but since they are not mutually exclusive, it is exceedingly difficult to pinpoint the exact function of distress calls in any given species. Theoretically, *S. bilineata* should be able to mob a predator because they are highly manoeuvrable [40]; however, their slight built and light weight [30] probably make mobbing attempts less effective than those of bigger bats [41–43]. We never observed mobbing behaviour towards the loudspeaker during our playback trials, but this could be caused by the absence of a real predator or a predator model. Alternatively, *S. bilineata* could approach distress calls to gain information about potential danger. In this scenario, high manoeuvrability would also be advantageous. The fact that *S. bilineata* exhibited phonotaxis only to conspecific, but not to heterospecific distress calls (*M. molossus* is a sympatric species that often occupies the same buildings as *S. bilineata*) could indicate that bats are more motivated to come to the aid of conspecifics instead of heterospecifics. However, again, bats might simply be more motivated to selfishly inspect potential danger announced by conspecifics instead of heterospecifics. As Carter *et al.* [23] rightfully pointed out, mere phonotaxis should not suffice as clear evidence for mobbing in bats.

*Saccopteryx bilineata*'s responsiveness to conspecific distress calls depended on the location in which they were broadcast (and, thus, probably on their perceived relevance): focal bats reacted to distress call playbacks broadcast within the day-roost and in close proximity to it, but not at a foraging site. This could mean that predation events close to their perennial day-roosts constitute a greater threat for *S. bilineata* than predation events in their fluctuating foraging grounds (and thus elicit more inspection and/or mobbing). Moreover, if mobbing was indeed the cause for the bats' approach behaviour, it would be more beneficial to mob in the vicinity of the day-roost, because *S. bilineata*'s stable group composition [30] would make reciprocal aid possible. When foraging, however, *S. bilineata* fly out of earshot from group members and do not use communal night-roosts [32].

In conclusion, our study shows that a socially roosting but solitarily foraging bat readily reacts to distress calls within the day-roost or in proximity to it but not at a nearby foraging site. Therefore, future studies on distress calls would benefit from taking the perceived social relevance of a given predation event into account, especially when comparing the production of or responsiveness to distress calls across species [11].

## Supplementary Material

ESM_dataset: This exel file provides raw data of the playback experiments and formatted data which were used in the statistical analysis.

## Data accessibility

The dataset supporting this article has been uploaded as part of the electronic supplementary material.
